# Axon Loss and Collagen Deposition Confirms Compression Neuropathy in the Ilioinguinal Nerve Resected From Primary Inguinal Herniorrhaphy Patients

**DOI:** 10.3389/jaws.2025.15658

**Published:** 2026-01-15

**Authors:** Robert Wright, Kiyrie Simons, Troy Sanders, Julia Wright, Troy Salisbury, Kseniya Shin, Donald Born, Anjali S. Kumar, Makena Horne, Rachel Daniel

**Affiliations:** 1 Cascade Hernia & Surgical Solutions at Meridian Surgery Center, Puyallup, WA, United States; 2 Elson S. Floyd College of Medicine, Washington State University, Spokane, WA, United States; 3 Uniformed Services University, Bethesda, MD, United States; 4 Western University of Health Sciences, Pomona, CA, United States; 5 Loma Linda University School of Medicine, Loma Linda, CA, United States; 6 Department of Statistics, University of Washington, Seattle, WA, United States; 7 Department of Pathology, Stanford University, Stanford, CA, United States; 8 Rosalind Franklin University of Medicine and Science, North Chicago, IL, United States; 9 Saint Louis University, St. Louis, MO, United States

**Keywords:** ilioinguinal nerve, inguinal hernia, compression neuropathy, axon loss, collagen fibrosis, Wallerian degeneration

## Abstract

**Introduction:**

Prior studies show pre-operative pain in primary inguinal hernia patients is associated with visible enlargement of the ilioinguinal nerve at the external ring. However, the ilioinguinal nerve has not been previously examined for evidence of axon loss in conjunction with inguinal hernia. This study investigates axon loss and collagen deposition in the ilioinguinal nerve as evidence of compression neuropathy associated with primary inguinal hernias.

**Methods:**

Ten male patients with visible ilioinguinal nerve enlargement during primary inguinal herniorrhaphies were enrolled in this prospective study. Resected nerve samples included the proximal (control), canal, and distal segments relative to the external ring. Epoxy resin sections were stained with toluidine blue to assess axon loss, and paraffin sections were stained with trichrome stains to evaluate collagen content.

**Results:**

Moderate to severe axon loss was observed in the canal and distal segments in 70% and 80% of patients, respectively. The canal segment demonstrated a significant increase in collagen content when compared to the proximal control (p < 0.02). Fascicular cross-sectional area increased significantly in canal and distal segments compared to control (p < 0.0058, p < 0.0098). The total nerve area of the canal segment was significantly smaller compared to the proximal control and distal segment (p < 0.006, p < 0.04).

**Discussion:**

In patients with primary inguinal hernias, the exposure of the ilioinguinal nerve within the canal segment can be associated with moderate to severe axon loss and increased fascicular areas due to collagen fibrosis, consistent with compression neuropathy.

## Introduction

Previous reports have described ilioinguinal nerve enlargement at the external inguinal ring in as many as 63% of patients with primary inguinal hernias [1]. Increased preoperative pain has been found to be correlated with increased myxoid content in the endoneurium and perineurium of ilioinguinal nerves [1]. Patients with visible enlargement of the ilioinguinal nerve were found to have significantly increased correlation with preoperative pain scores compared to patients without nerve enlargement [2]. In previous studies, 81% of patients found with enlarged ilioinguinal nerves were histologically confirmed to have increased myxoid material and perineural fibrosis consistent with compression neuropathy [3].

Compression neuropathy can occur as a peripheral nerve becomes constricted and the nerve becomes damaged due to prolonged physical contact with other tissues, such as fascia. Histologically, compression neuropathy is often associated with characteristic connective tissue changes including presence of myxoid content and fibrosis of the epineurium, perineurium and endoneurium within neural bundles [1]. Axons directly affected can exhibit demyelination and signs of Wallerian degeneration (i.e., generalized axon loss) depending on the length of time of exposure to pressure and degrees of response to the pressure, as found in other studies [4, 5]. This can become permanent damage as graded by Sunderland’s Classification of nerve injury [6].

Compression neuropathy has been defined in prior studies detailing the effects of compression on the median and ulnar nerves in the upper limbs. Possible compression neuropathy has not been well defined with respect to the ilioinguinal nerve. Clinically, compression neuropathy tends to present as pain or numbness and up to 20% of patients found to have a form of compression neuropathy never fully recover from its effects [7, 8].

To find evidence of compression neuropathy along the ilioinguinal nerve histologically, a trichrome stain is commonly used to show nerve structures including collagen and myelin. This allows the observer to visually separate collagen fibres of the extracellular matrix from other types of tissues such as muscle tissue and other epithelia [9]. The trichrome stain is an exceptionally useful tool for finding evidence of compression neuropathy since the stain detects changes in connective tissue, such as collagen, as seen in scarring [9].

A prior study using the trichrome stain to evaluate fascicular area, nerve cross sectional area, and collagen content in grossly enlarged ilioinguinal nerves found a narrowing in nerve fascicles within the inguinal canal, consistent with compression neuropathy caused by chronic pressure from hernia tissue [10]. This narrowing was associated with decreased fascicular area in the canal region, indicating significant nerve fibre degeneration likely due to ischemia from chronic pressure. Collagen levels remained uniformly distributed along the nerve, which suggested that fibrosis or extensive collagen degeneration was not the primary driver of the narrowing. Though the previous study emphasized structural aspects such as decreased fascicle size, there was no assessment for axon loss. This current study aims to investigate axon loss and collagen distributions and their respective relationships to compression neuropathy.

Although trichrome stain is useful for overall structure, it does not give sufficient detail to see myelinated axons. Axon loss can be seen histologically by embedding nerve tissue in epoxy resin then staining sections with toluidine blue. This stain has the capability of highlighting key features needed for detection of axon loss, in particular the myelin sheaths are intensely stained and can be used to assess the number of myelinated axons [11]. Loss of a significant number of axons within a nerve is considered enough evidence of compression neuropathy [12].

The ilioinguinal nerve has not been examined for axon loss and changes in collagen content using histology and neuropathological methods despite the common finding of enlarged ilioinguinal nerves in patients who undergo primary inguinal herniorrhaphies. A factor in the apparent enlargement of the ilioinguinal nerve may be directly attributed to compression neuropathy due to the inguinal hernia itself (i.e., the primary inguinal hernia has exerted direct pressure onto the ilioinguinal nerve over an extended period). Institutional Review Board (IRB) approved for the prospective study of male primary inguinal hernia patients undergoing open Lichtenstein repair. It was hypothesized that in enlarged ilioinguinal nerves harvested during primary inguinal hernia repairs, the trichrome and toluidine blue stains will demonstrate increased collagen content and axon loss, respectively, compared to the unaffected proximal ilioinguinal nerve.

## Methods

The Institutional Review Board (IRB) approved the prospective study of ten primary inguinal hernia males, who underwent open Lichtenstein repair. The IRB was approved because of previous research which indicated that pragmatic ilioinguinal nerve neurectomy during Liechtenstein repair can decrease pain without increasing paraesthesia [13]. Additionally, a recent body of literature suggests certain patients have histologic findings consistent with compression neuropathy [14].

**FIGURE 1:**
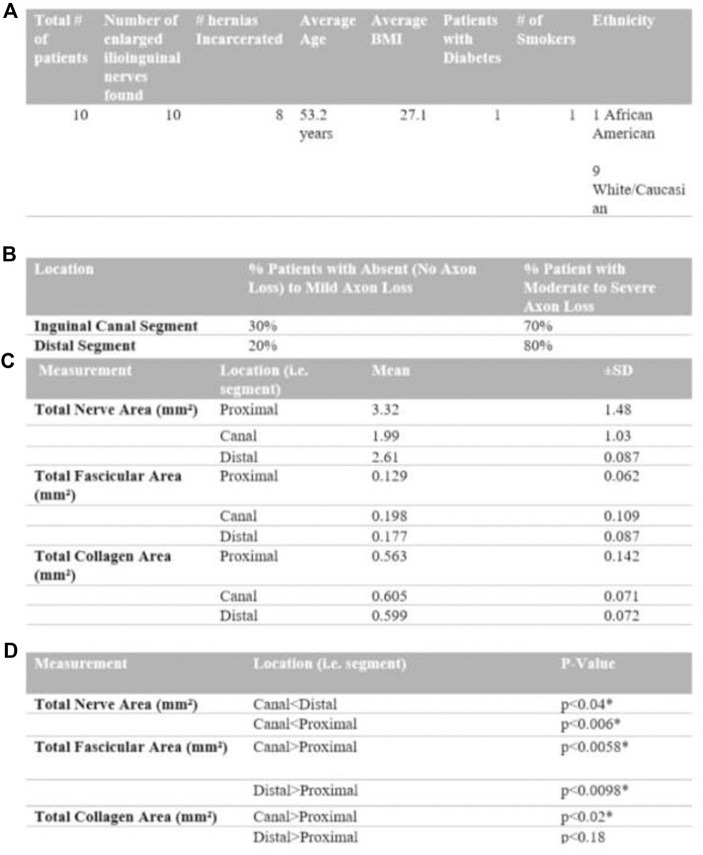
Composite Figure (from top to bottom as **A** to **D**) summarizing patient demographics (table **(A)**), toluidine blue stain qualitative grading (table **(B)**), collagen deposition on trichrome stain (table **(C)**) and segment comparison (table **(D)**).

**FIGURE 2:**
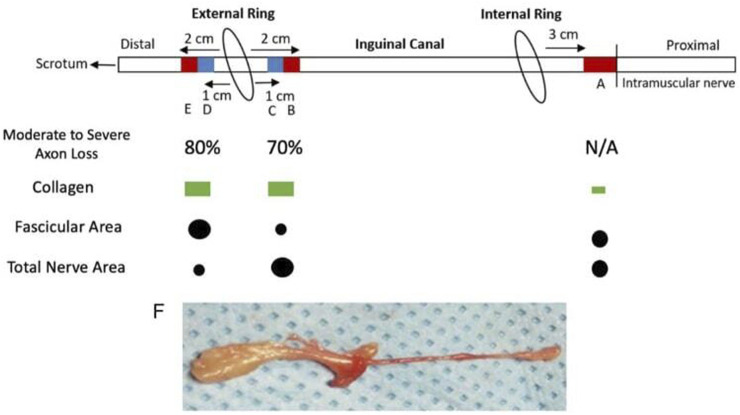
Illustration of Ilioinguinal Nerve and Resected Segments as it Progresses through the Inguinal Canal*. **(A)** Proximal segment not in contact with herniated tissue stained with Masson’s trichrome for collagen. **(B)** Inguinal canal segment proximal to external ring, stained with Masson’s trichrome for collagen. **(C)** Inguinal canal segment proximal to external ring, stained with toluidine blue for axons. **(D)** Distal segment to external ring, stained with toluidine blue for axons. **(E)** Distal segment to external ring, stained with Masson’s trichrome for collagen. **(F)** Representative whole ilioinguinal nerve specimen.

This study originally involved 30 patients, but due to the complexity of staining and requirement of a full dataset consisting of five samples per patient, only ten of the original 30 were viable for analysis. Figure 1A shows a summary of patient demographics. The ten patients who were studied were all male and all found to have grossly enlarged ilioinguinal nerves during primary inguinal herniorrhaphies (Figure 2F) and all had clinical preoperative inguinal pain as uncovered by the Carolina Pain score, filled out by these patients. To be included in the study the ilioinguinal nerve in the canal segment had to have less than half of the apparent diameter of the same ilioinguinal nerve at the external inguinal ring. Apparent fibrosis of the external inguinal ring fascia to the nerve did not exclude that nerve but rather supported its inclusion. If these findings were visually confirmed after neurectomy, the nerve was then included in the study. Eight of the patients had incarcerated inguinal hernias. The ten male patients possessed an average BMI of 27.1 and average age of 53.2 years. One patient had diabetes and one patient was an active cigarette smoker. One patient was African American, and nine patients were white. Several sections of the ilioinguinal nerve were taken. Three centimetres proximal to the internal inguinal ring, adjacent to the penetration of the ilioinguinal nerve through the internal oblique, a 1 cm section of nerve was harvested and underwent trichrome staining (Figure 2A). Two centimetres proximal to the external ring, a 1 cm section of nerve was also stained with trichrome (Figure 2B) and 1 cm proximal to the external ring another segment was stained with toluidine blue stain (Figure 2C). A 1 cm segment immediately distal to the external ring (distal ring segment) was also stained with toluidine blue (Figure 2D) and 1 cm distal to that segment, the most distal segment was also trichrome stained (Figure 2E).

Portions of the nerves were fixed in glutaraldehyde, embedded in epoxy resin (plastic), sectioned at 1 mm and stained with toluidine blue. Separate portions were fixed in formalin, embedded in paraffin wax, sectioned and stained with the trichrome stain. The stained sections were evaluated for the number of fascicles, the cross-sectional area of the nerve itself, the cross-sectional area of perineurium, the number of fascicles and the cross-sectional area of the fascicles. The nerve segments prepared with the trichrome stain were evaluated for collagen concentration. The toluidine blue stain segments level of neuron damage was graded as normal, mild, moderate, and severe.

### Trichrome Stain Analysis

After formalin fixation, the segments were paraffin-embedded, oriented, sectioned, and stained with modified Masson’s trichrome (collagen green, myelin red, and nuclei dark red brown). The stained sections were digitized [Aperio AT2, Leica Biosystems, USA] and analysed with Fiji ImageJ [Version 1.52n, NIH, USA]. Nerve cross-sectional area (mm^2^) was measured by manually drawing the region of interest (ROI) around the entire nerve. An additional ROI was drawn around the individual nerve fascicles (within the nerve sections, excluding the perineurium) and summed to determine total area covered. Using a filter customized to Masson’s stain, colour deconvolution in Fiji was applied to extract the green component of the original images. To quantify collagen content, total area (mm^2^) covered by the green component was measured against fascicle ROI total area. Results were analysed using a paired t-test with significance at p < 0.05.

### Toluidine Blue Stain Analysis

Following formalin fixation, segments were paraffin-embedded, reoriented, sectioned and stained with toluidine blue. This stain possesses a high affinity for acidic tissue components such as fatty acids contained in membranes and myelin sheaths [11]. The stained sections were digitized following preparation [Aperio AT2, Leica Biosystems, USA] and analysed using Aperio ImageScope [Aperio AT2, Leica Biosystems, USA]. The nerve cross-sectional area (mm^2^) was quantified by manually drawing around the interior and exterior of the fascicle boundary. The difference between the exterior and interior of the fascicular boundary was calculated and is defined as the perineurium. In addition to determining the area of the perineurium, the number of fascicles were also counted.

To determine the extent of axon loss, a qualitative myelin classification was used. This classification graded myelin within fascicles and included observable decreases in axon diameter, which can be indicative of Wallerian degeneration. The four classifications include normal, mild, moderate, and severe. Nerves were given a normal classification if there was little to no evidence of myelin damage or Wallerian degeneration (e.g., the blue highlighted myelin was shown collapsing into the intramembranous/cytoplasmic portion of the axons). Nerves were assigned a mild classification if there were a noticeable number of axons with evident myelin damage or Wallerian degeneration that affected a small number of axons. Nerves were designated a moderate classification if at least half of the axons possessed evidence of myelin damage. Nerves were granted a severe classification if a majority, or nearly all, axons had myelin damage or had signs of Wallerian degeneration.

## Results

### Toluidine Blue Stain

Figure 1B includes a summary of findings from the utilization of the toluidine blue stain. There were no proximal control segments prepared with the toluidine blue stain. Following utilization of qualitative myelin classification, the inguinal canal segment showed absent to mild axon loss in thirty percent of patients and moderate to severe axon loss in seventy percent of patients. The distal segment showed absent to mild axon loss in twenty percent of patients and moderate to severe axon loss in eighty percent of patients.

### Trichrome Stain

Figure 1C summarizes findings from comparisons between nerve segments analysed using the Trichrome stain Figures 1C,D. The trichrome stain facilitated measurement of total nerve area, nerve fascicle area, and collagen area of the distal, canal and proximal control segments of the ilioinguinal nerve for each of the ten patients enrolled. The total nerve area (mm^2^) (which includes extracellular matrix components, collagen and fascicles) of the canal segment was significantly smaller compared to the proximal control and distal segment (*p* < 0.006, *p* < 0.04), respectively. The neural fascicular cross-sectional area (mm^2^), however, was significantly larger at the canal and distal segment compared to the proximal control (*p* < 0.006, *p* < 0.01, respectively) with the canal segment being found to have a larger fascicular area than the distal segment.

**FIGURE 3:**
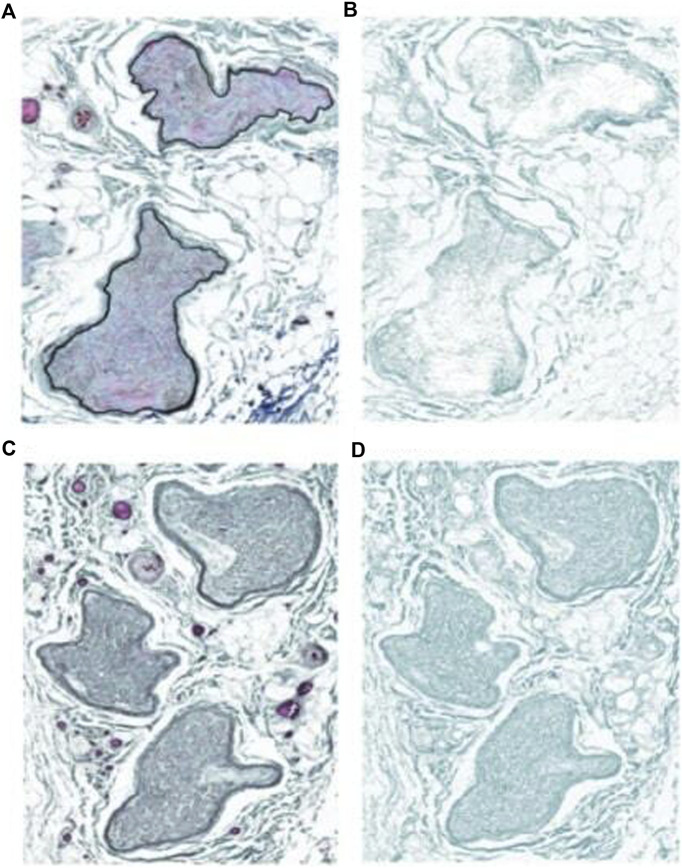
Images of Masson’s trichrome stain and collagen deposition on the ilioinguinal nerve. **(A)** Proximal sample. **(B)** Trichrome stain low collagen. **(C)** Inguinal canal sample. **(D)** Inguinal canal trichrome stain high collagen.

**FIGURE 4:**
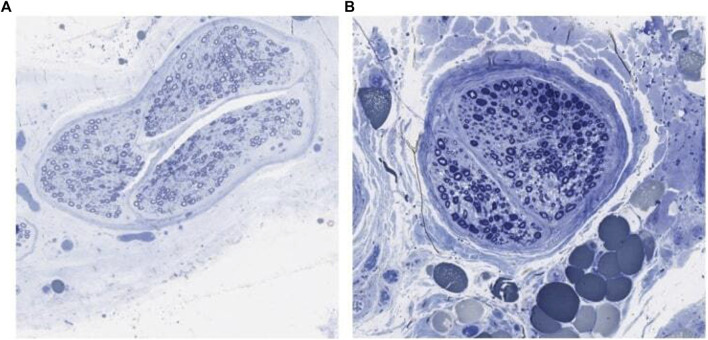
Toluidine blue stained ilioinguinal nerve showing axon loss. **(A)** Canal segment graded normal. **(B)** Canal segment graded severe axon loss.

The total collagen area (mm^2^) for the canal segment is larger in magnitude compared to the proximal control (*p* < 0.02). Figure 3 depicts the collagen staining. There was no significant difference in collagen area between the distal segment and the proximal control (*p* < 0.18). Therefore, compared to the proximal control, the canal segment of the ilioinguinal nerve shows a high degree of axon loss and smaller overall diameter in the setting of significantly higher collagen content within the fascicle (Figure 4).

## Discussion

In patients found to have apparent enlargement of the ilioinguinal nerve at the external inguinal ring during primary herniorrhaphy, compression neuropathy is likely to be present. This represents Sunderland class III irreversible nerve injury when present [6]. Histologically, the nerve shows evidence of collagen fibrosis in addition to Wallerian degeneration/axon loss, and excessive buildup of myxoid content. In this way, the ilioinguinal nerves gathered from these patients appear to have changes consistent with compression neuropathy. Anatomically, compression neuropathy occurs at locations where fascia or ligaments intercept nerves. Pressure exerted from fascia or ligaments can result in axon degeneration [15]. Experimentally, this is defined as a dose-response with both the amount of pressure and the time of compression being involved [16]. The exerted pressure on the nerve results in impairment of blood flow. The endoneurium does not have direct arterial or venous flow, so the diffusion of nutrients is impaired when pressure is exerted on the nerve as seen in a mini compartment syndrome [17].

The histology of compression neuropathy is well defined in the literature, both from animal studies [4, 17, 18] and human autopsy [5, 19, 20]. In compression neuropathy, at the site of compression, the nerve appears to be narrower. Distally, the nerve appears to have a larger diameter which is more consistent with the more proximal diameter. In inguinal hernias, the intra-abdominal pressure is transmitted and may intersect with the ilioinguinal nerve at the external inguinal ring, under the external oblique fascia. The enlargement of the ilioinguinal nerve and compression neuropathy occur most frequently when the external oblique is a sturdy structure and intersects with the ilioinguinal nerve at the external inguinal ring causing gross white thickening and fibrosis of both the external ring and the nerve [3]. Intra-abdominal pressure is normally 5–8 mmHg and increases substantially to 100 mmHg with cough and Valsalva maneuvers [21]. With a normal blood pressure, coughing could result in a substantial decrease in arterial blood flow to all structures within the herniated inguinal canal, including the ilioinguinal nerve [22, 23]. Since the trajectory of the nerve is quite variable, some patients will not experience that phenomena whereas others may experience it even with a relatively small hernia [3].

The discovery of changes associated with compression neuropathy (i.e., axon loss and collagen fibrosis) brings a new dimension to the understanding of not only preoperative hernia pain, but postoperative groin pain as well. Studies have demonstrated that patients who experience increased preoperative pain will have a higher likelihood of chronic postoperative pain [24–26]. Surgeons tend to blame technique; attorneys are happy to join in that chorus. However, the basic science now suggests that preexisting compression neuropathy is present in a subset of patients with inguinal hernias. With compression neuropathy in the carpal tunnel and also ulnar nerve compression neuropathy at the elbow, surgical relief is associated with 75%–80% complete normality in sensory and motor function; however, these operations are plagued with a significant proportion of patients who continue to have neurologic symptoms related to the presurgical compression neuropathy [27–30]. It can be speculated that the same phenomenon is occurring in postoperative inguinal hernia patients. When a surgeon performing an open inguinal hernia sees a size discrepancy between the ilioinguinal nerve distal to the external ring and proximal to the external ring, the withered proximal nerve represents compression neuropathy and can be easily visualized by the surgeon.

Understanding and avoiding chronic post-herniorrhaphy pain is the primary goal of the American Hernia Society [31]. The demonstration of compression neuropathy frequently occurring in the ilioinguinal nerve if confirmed by larger studies presents a quandary to the surgeon as to what is the best management for that compromised nerve. If left in place, compression neuropathy can resolve asymptomatically in many patients, however, a proportion of patients can be expected to suffer from chronic pain [8]. These nerves cannot be visually inspected with the laparoscopic approach, meaning that all laparoscopic repairs leave this in question. The treatment of post-herniorrhaphy pain by triple neurectomy has a relatively high success rate, particularly in patients whose pain seems to be neurogenic [31, 32]. The occurrence of compression neuropathy in the inguinal canal will require further study and may provide an explanation for postoperative pain states that have not previously been considered.

## Limitations

This is a small study and suffers from the typical problems of a small number of patients. It is not generalizable to the broader patient population of inguinal hernias as this exclusively was a study of males who demonstrated apparent enlargement of the ilioinguinal nerve at the external inguinal ring. Further studies involving females and confirmatory studies of a larger nature with attention to the toluidine blue staining in patients who are both symptomatic and asymptomatic may help further define our understanding of preoperative pain and possibly postoperative pain. Additionally, our original study size of thirty patients was significantly diminished by the inability to acquire analysable slides with the toluidine blue stain. Other stains may be utilized to broaden the understanding of the ilioinguinal nerve, including analysis of images gathered using electron microscopy [5].

## Conclusion

It can be concluded that, with the ten male patients involved in this study, the toluidine blue stain has successfully demonstrated evidence of axon loss and that the trichrome stain has demonstrated fibrosis in the ilioinguinal nerves removed from these patients, most profoundly in the canal segment. The ilioinguinal nerves from these patients exemplified histological traits consistent with compression neuropathy graded as a Sunderland Class III nerve injury [6].

## Data Availability

The original contributions presented in the study are included in the article/supplementary material, further inquiries can be directed to the corresponding author.
